# Asteroid Hyalosis: A Mimicker of Vitreous Hemorrhage on Point of Care Ultrasound: A Case Report.

**DOI:** 10.24908/pocus.v8i2.16391

**Published:** 2023-11-27

**Authors:** Eniola C Gros, Lauren R Mccafferty

**Affiliations:** 1 Department of Emergency Medicine, University Hospitals Cleveland Medical Center/Case Western Reserve University School of Medicine

**Keywords:** Diagnosis opthamology, ocular ultrasound

## Abstract

Ocular point of care ultrasound (POCUS) can help make timely recognition of multiple emergent ocular conditions and differentiate these from more benign conditions. While asteroid hyalosis (AH) is benign, it can easily mimic the more potentially serious vitreous hemorrhage on ocular POCUS, as both consist of numerous echogenic opacities within the vitreous with a classic “washing machine” appearance with eye movement. However, asteroid hyalosis particles tend to be more discrete, hyperechoic, scintillating, and seen throughout the vitreous. Knowledge of this mimic and ability to recognize the subtle sonographic differences can help differentiate these disease processes, which can influence management and potentially disposition.

## Background

Point of care ultrasound (POCUS) is often utilized in the emergency department (ED) to quickly evaluate for several potentially vision-threatening pathologies. This includes, but is not limited to, retinal detachment (RD), foreign body, lens dislocation, posterior vitreous detachment (PVD), and vitreous hemorrhage (VH) 1, 2. A potential mimicker of VH in particular is asteroid hyalosis, a relatively rare, benign degenerative condition that often has little impact on vision 3, 4. Similar to VH, POCUS findings of asteroid hyalosis consist of mobile hyperechoic opacities within the vitreous 5, 6, 7 . Being able to recognize the subtle differences between the two is important, as the management of each is quite different.

We present a case of a patient who presented to the ED for painless monocular vision changes. Ocular POCUS revealed numerous distinct, mobile, hyperechoic opacities throughout the vitreous and was initially thought to be VH. However, on closer inspection and with ophthalmologic evaluation, the patient was diagnosed with asteroid hyalosis and did not require additional ophthalmologic management.

## Case Report

The patient was a fifty-nine year-old female with a history of hypertension, hyperlipidemia, coronary artery disease status post percutaneous coronary intervention, chronic obstructive pulmonary disease and fibromyalgia who presented to the ED with right-sided facial pain and intermittent blurred vision. She stated her symptoms began gradually while grocery shopping the prior evening and had been persistent since. She denied a headache, foreign body sensation, flashes of light, floaters, double vision, vision loss, pain with extraocular movements, or abnormalities of the surrounding skin.

Physical examination revealed pupils that were equal, round, mid-range in size, and reactive to light bilaterally. Extraocular movements were intact. There were no keratotic lesions, hyphema, or conjunctival injection noted. There was tenderness to light touch over the right periorbital region, cheek and nose without any skin lesions, erythema, or swelling. Intraocular pressure was within normal limits. Visual acuity was 20/40 in the right eye, 20/30 in the left eye. Intraocular pressure was within normal limits. A neurologic examination further revealed fluent speech, normal strength and sensation in all four extremities, and no facial asymmetry.

**Figure 1:**
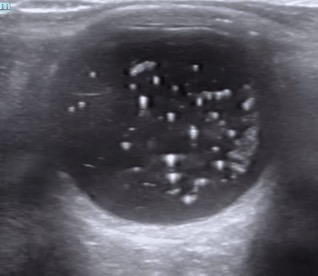
Transverse view of the affected eye demonstrates distinct hyperechoic opacities within the vitreous body consistent with asteroidhyalosis.

On POCUS of the right eye, there was no evidence of RD. However, within the vitreous there were numerous hyperechoic foci which were mobile with a dynamic exam. While it had the classic “washing machine”^5^ appearance of VH, the particles were more echogenic and distinct than what is typically seen with VH (Figure 1, Video S1). Ophthalmology was consulted given the patient’s presenting symptoms and abnormal ocular ultrasound findings. Dilated fundoscopic examination confirmed the diagnosis of asteroid hyalosis. No other acute pathology was noted, other than possible early herpes zoster ophthalmicus. Outpatient management was deemed appropriate, so she was discharged home with a prescription for antivirals and advised to follow up with ophthalmology later that week. She did not require follow up for the asteroid hyalosis alone.

## Discussion

Asteroid hyalosis, named for resembling “stars in a night sky”, is a benign degenerative ocular condition resulting in calcium, phosphate, and lipid deposits varying in size within the vitreous body 3. Increasing age and male sex are the most significant risk factors for asteroid hyalosis 4. Systemic comorbidities, such as diabetes, hyperlipidemia, and hypertension, have been reported to be associated with asteroid hyalosis 8; however, when adjusted for age and sex, this association appears to lack significance 9.

 Asteroid hyalosis is rarely symptomatic unless severe or if concurrent ocular pathology, including cataracts, vitreous hemorrhage, or diabetic retinopathy, is present. While often an incidental finding, asteroid hyalosis can confound retinal or fundoscopic imaging due to its numerous vitreous opacities 3, 8. While often detected by a comprehensive ophthalmologic examination, asteroid hyalosis can also be detected with POCUS, a readily available diagnostic tool for emergency physicians.

**Figure 2:**
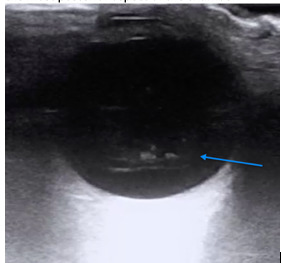
Transverse view of the affected eye demonstrating vitreous hemorrhage, characterized by echogenic blood layering posterior in the vitreous body (blue arrow). The more hyperechoic areas represent clotted blood.

A POCUS assessment for asteroid hyalosis is similar to that of RD, PVD, or VH, which is well described in the ultrasound literature 1, 3, 4, 5, 10, 11. Visualization of these conditions is often optimized when the gain is increased. Asteroid hyalosis most closely mimics vitreous hemorrhage (Figure 2), as both consist of numerous echogenic opacities within the vitreous with a classic “washing machine” appearance with eye movement. However, asteroid hyalosis particles tend to be more discrete, hyperechoic, scintillating, and seen throughout the vitreous, whereas vitreous hemorrhage particles tend to be more heterogeneous and layer in the most posterior aspect of the chamber 1, 3.

Given its benign, degenerative nature, asteroid hyalosis rarely requires any particular treatment and non-emergent ophthalmologic follow-up is appropriate. It often does not require further ophthalmologic workup unless other pathology is suspected, or significant vision loss occurs. 

## Conclusion

Emergency physicians can use POCUS to promptly evaluate for several time-sensitive, vision-threatening ocular conditions. While relatively rare, asteroid hyalosis is a benign condition that can easily mimic VH, RD, or other concerning pathology that typically warrants urgent or emergent ophthalmologic evaluation. Knowledge of this mimic and ability to recognize its subtle sonographic features can be useful in differentiating from other pathologies, communicating with ophthalmology, and influencing management and disposition.

## Disclosures

The authors declare that they have no known competing conflicts interests or personal relationships that could have appeared to influence the work reported in this paper. 

## Patient Consent

Patient consent was obtained by the authors in addition to approval from the hospital ethics department.

## Supplementary Material 

 Video S1 Asteroid hyalosis. Numerous hyperechoic foci within the vitreous which are mobile with a dynamic exam.
